# Varicella-Zoster meningitis with normal CSF cellularity: A rare case report

**DOI:** 10.1016/j.idcr.2022.e01484

**Published:** 2022-03-25

**Authors:** Bana Sabbagh, Mohammed Najdat Seijari, Mhd Kutaiba Albuni, Munsef Barakat, Gamal B. Alfitori

**Affiliations:** a Damascus University, Damascus, Syria; b Department of Internal Medicine, Hamad General Hospital, Hamad Medical Corporation, Qatar

**Keywords:** Aseptic meningitis, Varicella-Zoster, CSF analysis

## Abstract

Varicella-zoster virus (VZV) meningitis is a one of the manifestations of VZV reactivation which usually presents with fever, headache, and is sometimes preceded by a dermatomal vesicular rash. CSF analysis is the cornerstone investigation in helping to identify the causative organism or in orienting the physician toward a possible agent. CSF analysis in Viral meningitis usually reveals lymphocytic pleocytosis. However, normal CSF analysis with viral meningitis -despite being rare- has been reported especially with bacterial causes. Therefore, a first CSF puncture cannot rule out infection and a second one might be warranted if there is still high suspicion of viral meningitis with no diagnosis made by PCR. Here we present a case of an 89-year-old female who had signs and symptoms of meningitis with normal CSF analysis. However, polymerase chain reaction (PCR) was positive for VZV. The patient was treated accordingly, and she recovered fully.

## Introduction

Varicella-zoster virus (VZV) presents with many neurological complications after reactivation, the most common being a dermatomal vesicular eruption that is usually preceded by prodromal pain, itching, or tingling. Meningitis in immunocompetent patients is considered a known complication of VZV. It often manifests with a headache, fever, and sometimes a rash that may present 3–5 days after meningitis. This is along with lymphocytic pleocytosis in CSF analysis [1]. However, some cases in the literature have recently described viral meningitis with no pleocytosis such as Enterovirus and Herpes Simplex Virus [2], [3]. We hereby present a case of VZV meningitis in an elderly patient along with the dermatomal rash and normal CSF findings.

## Case presentation

An 89-year-old female patient with a history of hypertension and asthma presented with a severe left-sided occipital headache that has started three days prior to hospitalization. The headache was persistent, very severe in intensity, did not respond to analgesics. The patient denied any vomiting, fever, photophobia, or decrease in vision. Regarding the patient’s past medical history, it should be noted that her asthma was well controlled by a salbutamol inhaler as needed, hence no ED visits nor hospitalizations for asthma were reported. She was not on systemic steroids and didn’t receive pulse steroid therapy before. Upon further review of her vaccination history, she has not received the varicella vaccine before.

On physical examination: the patient was alert and oriented, had left jaw angle tenderness, left occipital and temporal scalp tenderness, with no rash overlying the temporo-occipital area. She had periorbital swelling after exposure to diclofenac sodium, which she took over the counter as topical treatment one day before admission, even though the patient has documented a history of allergy to diclofenac potassium.

There were two noticeable 1 cm erythematous papules over the left eyelid only. However, on the second day of admission, the patient developed a papulovesicular rash on the left side of the forehead and upper eyelids. Moreover, Kernig and Brudzinski signs were negative but there was limited mobility of the neck with tenderness.

Vital signs on presentation: Temp Oral: 36,7 °C, Heart Rate: 91 beat/min, Resp Rate: 17 breath/min, systolic blood pressure: 154 mmhg, diastolic blood pressure: 77 mmhg, oxygen saturation: 99% on room air. Laboratory results on presentation summarized in (Table 1). The patient was assessed by an ophthalmologist for left eyelid swelling and rash, and he mentioned a vesicular rash over the eyelid that does not involve lid margins or conjunctiva. The cornea was clear with no staining, with dense nuclear cataract, and no signs of zoster ophthalmicus were appreciated.

**Table 1:** Summarizes blood lab test at the day of admission.

Lab Test	Value	Normal range
White Blood count (WBC)	12.8	(4–10) * 10^3^/µl
Hemoglobin (HgB)	13.8	(12–15) g/dl
Platelets (PLTs)	380	(150–400)* 10^3^/µl
Absolute Neutrophils count	3.8	(2–7) * 10^3^/µl
Absolute lymphocytes count	7.5	(1–3) * 10^3^/µl
Urea	3.5	(2.5–7.8) mmol/L
Creatinine	62	(44–80) µmol/L
Sodium	136	(133–146) mmol/L
Potassium	3.9	(3.5–5.3) mmol/L

**Fig. 1:**
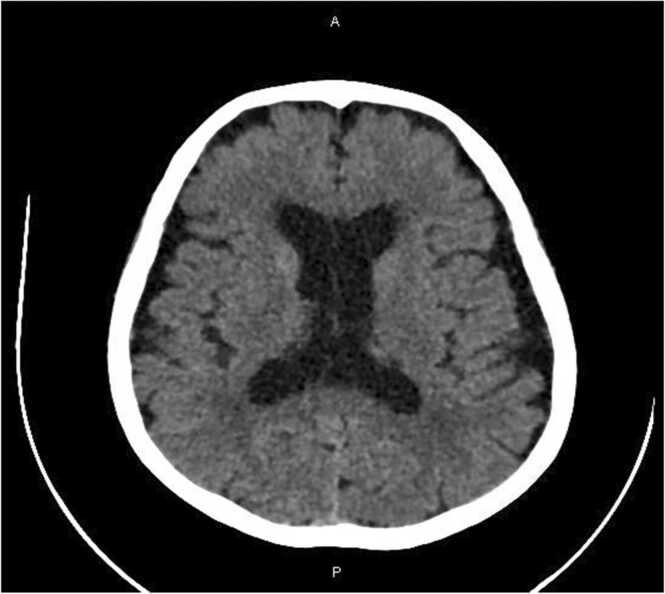
CT head: showed Age-related parenchymal involutional changes including dilated ventricular system.

Head CT without contrast (Fig. 1) showed Age-related parenchymal atrophic changes, otherwise, the study was unremarkable. Head MRI, MRV, MRA were also done, and they showed age-related changes along the cerebral sulci and ventricles, extensive periventricular and deep white matter ischemic gliotic changes, and few chronic lacunar infarctions were seen. Intracranial arteries and Dural sinuses were unremarkable with no radiologic evidence of arterial stenosis, aneurysm, or venous thrombosis. Ultrasound Doppler of both temporal arteries showed normal caliber arteries with normal wall thickness. The right artery wall measures 2.0 mm and the left measures 1.8 mm. Normal PSV and flow patterns were noted on both sides, overall, the findings were not compatible with temporal arteritis.

CSF analysis results showed Positive VZV PCR with normal cellularity, the results are summarized in (Table 2). VZV serology done retrospectively two days after the lumbar puncture revealed positive IgG antibodies in the serum with a simultaneous absence of IgM, no titters were available as the assay used in our department microbiology lab shows only qualitative results, and no assay for the presence of antibodies of VZV in the CSF was done.

**Table 2:** Summarizes CSF analysis.

Lab test	Value	Normal range
WBC (wight blood cell)	1	(0–5)/µL
RBC (Right blood cell)	0	(0–2)/µL
Glucose	4.05 Blood Glucose was 7.6	(2.22–3.89) mmol/L
Protein	0.44	(0.15–0.45)
Oligoclonal bands	Negative	
IgG index	0.5	(0.3–0.6)
Latex Angulation for Cryptococcus	Negative	
Tuberculosis (TB) PCR	Negative	
Gram stain and Culture	No Growth	
Cytomegalovirus (CMV) PCR	Negative	
Human Herpes Virus (HHV-6) PCR	Negative	
Herpes Simplex Virus (HSV-1 and 2) PCR	Negative	
Enterovirus PCR	Negative	
Varicella-Zoster Virus PCR	Positive	

Treatment: The patient did not receive empirical antimicrobial therapy prior to lumbar puncture because her illness was indolent in onset and did not have any obvious signs of bacterial infection. After LP, she was commenced on intravenous Acyclovir 10 mg\kg (670 mg) every eight hours due to the nature of her headache and the vesicular rash she developed, whilst waiting for CSF results. After confirming the diagnosis by PCR, the treatment was continued for 14 days. During her stay, her headache was controlled with oral tramadol. The initial dose was 50 mg three times per day, but the requirements decrease to once daily by the end of her stay.

Follow up: The patient got better, her hospital course was unremarkable, she completed her acyclovir course and was discharged with no pain and improved rash.

Two weeks after discharge, a follow up by the general medicine clinic confirmed that the patient was getting better regarding her skin rash, however, she was suffering from bouts of burning like pain in the left periorbital area, therefore, she was started on oral gabapentin 300 mg three times a day for postherpetic neuralgia management. Two weeks later in a subsequent medicine clinic follow-up, the patient reported improvement of the burning pain, no rash was seen on examination, and her gabapentin dosage was reduced to 300 mg once a day.

## Discussion

VZV is one of the alpha-herpes viruses that usually cause mild to moderate presentation with disseminated vesicular rash in the primary infection. After reactivation VZV can cause a wide range of neurologic diseases, with herpes zoster and post-herpetic neuralgia being the most frequent. Meningitis is a more severe manifestation of VZV reactivation [4], [1]. VZV meningitis is more prominent in immunocompromised states and older age patients. Patients mostly present with headaches, fever, lethargy, and sometimes rash that usually presents several days after the local pain [1]. Our patient presented with a severe left occipital headache and developed a rash six days after the onset of the headache.

After suspecting meningitis, initial CSF data (WBC, glucose, and protein) may help guide clinicians before a microbiological workup is done. CSF findings of lymphocytic pleocytosis usually predict a viral infection. While increased CSF lactate and decreased glucose ratio are useful predictors of bacterial infections [5]. WBC count in VZV meningitis is usually around 302 cells/mm^3^ [6]. Our patient’s CSF results showed no pleocytosis with normal glucose and protein.

Several studies have recently discussed cases of central nervous system infection with normal CSF cellularity. A recent systematic review found that most of the cases mentioned in the literature across all age groups were bacterial (99 of 124). Whereas viral and fungal infections caused infection with normal cytology at a lower rate [7]. The absence of pleocytosis has been described with several viruses, but not well described in VZV. Whole-brain irradiation was associated with normal CSF cytology results as discussed by Jakob and his colleagues in three patients with HSV encephalitis [2]. Additionally, a recent study that discussed the frequency of normal cerebrospinal fluid protein level and leukocyte count in enterovirus meningitis showed that out of 46 infants diagnosed with Enteroviral meningitis 13% had both normal protein and leukocyte count in their CSF [3]. Another study demonstrated that Enterovirus caused normal CSF cellularity in 68–77% of the neonates diagnosed with meningitis [8]. A case of an immunocompetent 23-year-old adult along with a 75-year-old patient who had HSV encephalitis but normal CSF cytology was also reported by Oğuz et al. [9]. HSV has also presented in 28 immunocompetent females with encephalitis but normal CSF cytology [10].

The cases reported were diagnosed by PCR which is the gold standard test in this case, as the overall percentage of positive initial CSF PCR for viral causes was 100% in the systematic review [7].

Most cases reported were either infants and neonates, elderly or immunocompromised. Our case supports these findings as the patient was 89 years old with hypertension and asthma. One may argue elderly populations are not fully immune component due to the phenomenon called Immunosenescence. This phenomenon is considered a part of normal aging, but it should be noted that different elderly people have varying degrees of it, and quantifying it currently is not a present option. Elders with significant immunosenescence usually have prolonged or frequent hospitalizations due to different infections that are targeted by cellular or humoral immune responses, are at increased risk of malignancy, and have decreased response to vaccines [11]. However, we can infer that this patient despite her age is still of good immune function due to lack of hospitalizations due to community-acquired or opportunistic infections till the time of presentation in the described case, and no history of previous or current malignancy is noted till now.

Yun et al. demonstrated that the proportion of CSF pleocytosis decreased significantly with age, as well as with the shorter duration between onset of symptoms and CSF puncture, and low peripheral white blood cell count [8]. This effect could be even more prominent in the immunocompromised. However, our patient’s labs on admission showed leukocytosis and elevation of lymphocytes (WBC 12.8 and lymphocytes were 7.5), with mildly elevated CRP (27 mg\l) which can indicate that the patient is mounting an immune response against this current infection.

We would also like to highlight that reactivation of VZV, as alluded to by the VZV serology, can result in aseptic meningitis. This was similarly reported by Platanaki et al. who described a case of VZV meningitis in a 56-year-old immunocompetent male, but unlike their case, our patient CSF analysis was completely normal [12]. Detecting VZV in CSF can sometimes be due to subclinical reactivation of VZV and this phenomenon has been described extensively in the human immunodeficiency virus (HIV) population. Birlea and Arendt in their study of different viruses’ reactivation in the CSF in HIV patients found out 28 HIV positive individuals who had evidence of VZV reactivation by intrathecal antibody synthesis. 27 out of those 28 individuals were completely asymptomatic. On the contrary, our patient had classical symptoms of severe headache in addition to the rash thus favoring the presence of clinical infection rather than subclinical reactivation [13].

The importance of early diagnosis and definition of the etiology of meningitis, whether bacterial or viral, lies in the fact that it is crucial for treatment decisions. Viral meningitis cannot be distinguished easily from other causes of central nervous system (CNS) infection; therefore, a rapid diagnosis is important for the appropriate management and infection control. Early diagnosis helps in decreasing unnecessary use of antibiotics especially if the presentation is more acute and antibiotics may be started empirically, hospital stay length as starting specific treatment such as Acyclovir lowers risks of complications, and it also helps in avoiding the costs of further investigations [14]. That’s why it is important to conduct powerful studies that reassess the diagnostic yield of CSF analysis in order to help guide clinicians more towards decision making. While emphasizing at the same time on maintaining a suspicion of CNS infection regardless of normal CSF cytology, especially in highly symptomatic patients. A repeat CSF puncture can be difficult due to the invasive nature of the test. Nevertheless, we emphasize that a single lumbar puncture with normal CSF cytology cannot rule out meningitis. And if no diagnosis was made with culture or PCR, physicians should consider repeating the test, especially If an infection is suspected clinically or the patient continues to deteriorate [15], [7].

## Ethical approval

Aprroved.

## Consent

Written consent is available upon request.

## Sources of funding

No Source of funding.

## CRediT authorship contribution statement

**Bana Sabbagh:** Manuscript writing, literature review, and approval of the final manuscript. **Mohammad Najdat Seijari:** History and physical, manuscript writing and editing, case identification and conceptualization, literature review. **Mhd Kutaiba Albuni:** manuscript writing and editing and corresponding author. **Munsef Barakat:** History and physical, case follow up. **Gamal B Alfitori:** Case selection, case identification and Conceptualization, obtain informed written consent, prescribing medicine, clinical follow up.

## Funding

Open Access funding provided by the Qatar National Libray.

## Declaration

We declare no conflict of interest.

## Conflicts of interest

No Conflict of interest.

## References

[1] Ihekwaba U.K., Kudesia G., McKendrick M.W. (2008). Clinical features of viral meningitis in adults: significant differences in cerebrospinal fluid findings among herpes simplex virus, varicella zoster virus, and enterovirus infections. Clin Infect Dis Publ Infect Dis Soc Am.

[2] Jakob N.J., Lenhard T., Schnitzler P., Rohde S., Ringleb P.A., Steiner T. (2012). Herpes simplex virus encephalitis despite normal cell count in the cerebrospinal fluid. Crit Care Med.

[3] Landry M.L. (2005). Frequency of normal cerebrospinal fluid protein level and leukocyte count in enterovirus meningitis. J Clin Virol Publ Pan Am Soc Clin Virol.

[4] Granerod J., Ambrose H.E., Davies N.W., Clewley J.P., Walsh A.L., Morgan D. (2010). Causes of encephalitis and differences in their clinical presentations in England: a multicentre, population-based prospective study. Lancet Infect Dis.

[5] Dittrich T., Marsch S., Egli A., Rüegg S., De Marchis G.M., Tschudin-Sutter S. (2020). Predictors of infectious meningitis or encephalitis: the yield of cerebrospinal fluid in a cross-sectional study. BMC Infect Dis.

[6] Lee G.-H., Kim J., Kim H.-W., Cho J.W. (2021). Herpes simplex viruses (1 and 2) and varicella-zoster virus infections in an adult population with aseptic meningitis or encephalitis. Medicine.

[7] Troendle M., Pettigrew A. (2019). A systematic review of cases of meningitis in the absence of cerebrospinal fluid pleocytosis on lumbar puncture. BMC Infect Dis.

[8] Yun K.W., Choi E.H., Cheon D.S., Lee J., Choi C.W., Hwang H. (2012). Enteroviral meningitis without pleocytosis in children. Arch Dis Child.

[9] Avkan Oğuz V., Yapar N., Sezak N., Alp Cavuş S., Kuruüzüm Z., Sayiner A. (2006). Two cases of herpes encephalitis with normal cerebrospinal fluid findings. Mikrobiyol Bul.

[10] Ahmed R., Kiani I.G., Shah F., Najeeb-ur-Rehman R., Ehsan-ul-Haq M. (2013). Herpes simplex encephalitis presenting with normal CSF analysis. J Coll Phys Surg Pak.

[11] Ongrádi J., Kövesdi V. (2010). Factors that may impact on immunosenescence: an appraisal. Immun Ageing.

[12] Platanaki C., Leonidou L., Siagkris D., Giannopoulou I., Paliogianni F., Velissaris D. (2021). Varicella-zoster virus aseptic meningitis: an atypical presentation in an immunocompetent male patient. Oxf Med Case Rep.

[13] Birlea M., Arendt G., Orhan E., Schmid D., Bellini W., Schmidt C. (2011). Subclinical reactivation of varicella zoster virus in all stages of HIV infection. J Neurol Sci.

[14] Dawood N., Desjobert E., Lumley J., Webster D., Jacobs M. (2014). Confirmed viral meningitis with normal CSF findings. BMJ Case Rep.

[15] Kindley A.D., Harris F. (1978). Repeat lumbar puncture in the diagnosis of meningitis. Arch Dis Child.

